# Acute kidney injury following ingestion of plate developer (sodium metasilicate): a case report

**DOI:** 10.1186/s13104-016-2225-x

**Published:** 2016-08-22

**Authors:** B. G. A. Rathnamali, G. Samarajiwa, D. D. K. Abeyratne, G. N. D. Perera, S. B. Gunatilake

**Affiliations:** 1 University Medical Unit, Colombo South Teaching Hospital, Kalubowila, Colombo, Sri Lanka; 2 Department of Medicine, University of Sri Jayewardenepura, Nugegoda, Sri Lanka

**Keywords:** Plate developer, Acute kidney injury, Sodium metasilicate

## Abstract

**Background:**

Plate developer is a chemical used in the printing industry and is a corrosive alkaline agent containing sodium metasilicate as the main substance. Plate developer poisoning is rare. Literature search revealed only a single case report of fatal sodium metasilicate poisoning (Z Rechtsmed 94(3):245–250, 1985). There are no reports of acute kidney injury related to ingestion of sodium metasilicate containing substances.

**Case report:**

A 52-year-old Sri Lankan male with a history of hypertension and affective disorder presented following ingestion of about 150 ml of plate developer solution. He developed severe upper airway obstruction due to laryngeal edema and underwent tracheostomy. While in the ward he developed features of acute kidney injury with high serum creatinine levels and persistent hyperkalemia which necessitated temporary haemodialysis. Because of the corrosive effect, he developed severe inflammation of the upper gastro intestinal tract with narrowing of esophagus and pyloric region, requiring feeding jejunostomy. He died while waiting for the surgery for pyloric stenosis.

**Conclusions:**

Acute kidney injury is a potential treatable complication of plate developer poisoning other than its complications related to corrosive effects. Regular monitoring of renal functions in such a patient would be useful for early recognition of acute kidney injury.

## Background

Plate developer is a corrosive alkaline agent which is used as an image developer for positive working thermal plates in printing industry. It is composed of sodium metasilicate, a proprietary modifier and water and poisoning with this agent is rare. Sodium metasilicate is the main constituent as well as the most hazardous agent. No reported cases were found of acute renal failure associated with ingestion of sodium metasilicate [1].

## Case presentation

A 52-year-old Sri Lankan male was admitted to hospital 30 min after ingesting about 150 ml of PD8 (plate developer) solution. He had a history of hypertension and affective disorder with a past history of suicide attempts. However, he was not on treatment for either condition regularly and for the 5 months preceding this admission, had not taken any medication. Poisoning center was contacted immediately for advice. Gastric lavage or activated charcoal was not instituted as the poison was a corrosive substance.

We managed him with fluid resuscitation (using 0.9 % NaCl solution), soon after admission. Within first hour of admission, patient developed stridor and hypoxia and an emergency tracheostomy was done. In next 48 h we managed him with intravenous dexamethasone 4 mg 8 hourly, nebulized adrenalin 4 hourly, salbutamol nebulization 2 hourly, and intravenous omeprazole 40 mg twice daily. Blood pressure was managed with oral nifedipine and prazosin and the systolic pressure remained above 110 mmHg and he did not become hypovolemic or hypotensive. A nasogastric tube was inserted to facilitate removal of gastric material, and 650 ml of altered blood was drained. His baseline serum creatinine value was not known, but it rapidly deteriorated over next 72 h (135.8 µmol/l at 2 h, 241 µmol/l at 24 h and 650 µmol/l at 72 h) and he developed hyperkalemia (7.2 mmol/l). It was initially managed with insulin-dextrose regime and calcium resonium. However serum potassium level remained persistently above 6 mmol/l. He remained oliguric for next 48 h despite adequate hydration and developed peripheral oedema and eventually pulmonary edema necessitating hemodialysis. His liver enzymes remained normal (aspartate transaminase 37 U/L, alanine transaminase 25 U/L) and there was no evidence of ischemic or toxic hepatitis. Urine full report showed no active sediments (pus cells 5/HPF, red cells nil, albumin nil, sugar nil, red cell casts nil). Chest radiography showed pulmonary edema. Haemodialysis was initiated on the 4th day of admission and repeated for the next 2 days. After the 4th haemodialysis serum potassium level became normal (4.7 mmol/l) and urine output improved. Serum creatinine declined steadily during this period and haemodialysis was stopped after the 5th dialysis. After 1 month at the time of discharge his serum creatinine level was near normal (115 µmol/l). During the hospital stay, patient developed intolerance to nasogastric feeding. Upper Gastro-intestinal endoscopy performed on day 20 revealed severe inflammation and narrowing of the lumen of esophagus, ulceration of the stomach and upper duodenum and narrowing of the pyloric opening. A feeding jejunostomy was done for enteral feeding on day 21. Patient also required blood transfusion to correct anaemia. Olanzapine and fluoxetine was started by psychiatrists for severe depression. Three months after discharge the jejunostomy was blocked and total parenteral nutrition was resorted to. He died while waiting for surgery to correct the pyloric stenosis from complications related to malnutrition.

## Discussion

Plate developer poisoning is rare. This is to our knowledge the first case report of acute renal failure following ingestion of plate developer. Sodium metasilicate is a constituent of various products and used for various purposes such as fireproofing mixtures, in laundry, for metal and floor cleaning, for de-inking recycled paper products in the pulp and paper industry, in washing carbonated drink bottles, in insecticides, fungicides, and antimicrobial compounds, and as a chemical intermediate for silica gel catalysts, an additive in soaps and synthetic detergents, an ingredient in adhesives, a bleaching aid. Combined with other salts such as sodium bicarbonate, it can be applied to aluminum as a paint stripper [2].

Toxicity of the sodium metasilicate depends on the concentration, the silica to alkali ratio, the sensitivity of the exposed tissue, and the length of exposure [3]. Soluble silicates can induce effects ranging from irritation to corrosion. Sodium metasilicate can produce caustic burns (i.e., colliquative necrosis) and induce hypocalcaemia by binding to calcium. In an animal study, oral administration of sodium metasilicate produced ulceration and bleeding in the stomach, duodenum and small intestine. Oral dosing of the 20 % solution caused dyspnea, and nasal discharge. Higher doses caused death [3].

### Sodium metasilicate associated renal injury

Intraperitoneal injection of nonahydrate form of metasilicate to guinea pigs has shown to produce silicaceous deposits in the kidney tubules. This effect was also demonstrated in another study in which dogs were given sodium metasilicate orally. Unspecified damage to kidneys, ureters, bladder, gastrointestinal tract, and the lungs were observed. When it was given orally to rats, slight degenerative changes in the epithelia of the renal tubules were observed [3].

Available human data also showed that oral administration of sodium metasilicate (TDLo = 1 mL/kg; 217 mg/kg) produced changes in kidney tubules, hematuria and caused nausea or vomiting [3]. But we did not find any reported cases of acute renal failure. Ultra sound study of kidney, ureter and bladder performed on the third day of admission showed normal kidney size with features of acute kidney injury and no evidence of obstructive uropathy. His creatinine level rose rapidly within few days with features of acute renal failure. His blood pressure remained high and it was managed with nifedipine and prazosin and lowest recorded blood pressure was 110/70 mmHg. There was no period of hypovolemia and hypotension. We did not use any nephrotoxic medications for the management of this patient. We could not perform a renal biopsy at that stage as patient was not stable. At the time of discharge creatinine was 115 µmol/l and it remained same after 3 months of follow up. It is likely that he had developed acute kidney injury due to direct toxicity of metasilicate on the kidneys.

## Conclusions

Clinicians should be aware that in addition to the corrosive injuries to gastrointestinal tract, acute renal failure can occur following ingestion of sodium metasilicate containing compounds such as plate developer. Therefore, acute renal toxicity should be anticipated in patients with plate developer toxicity and their renal functions should be monitored closely.

## Abbreviations

Ml: milliliter
PD8: plate developer 8
Mg: milligram
TDLo: lowest published toxic dose
